# Functional Rescue of Inactivating Mutations of the Human Neurokinin 3 Receptor Using Pharmacological Chaperones

**DOI:** 10.3390/ijms23094587

**Published:** 2022-04-21

**Authors:** Ross C. Anderson, Sharika Hanyroup, Yong Bhum Song, Zulfiah Mohamed-Moosa, Iman van den Bout, Alexis C. Schwulst, Ursula B. Kaiser, Robert P. Millar, Claire L. Newton

**Affiliations:** 1 Centre for Neuroendocrinology, Faculty of Health Sciences, University of Pretoria, Private Bag X323, Pretoria 0031, South Africa; sharika.hanyroup@gmail.com (S.H.); zulfiah.mohamedmoosa@up.ac.za (Z.M.-M.); iman.vandenbout@up.ac.za (I.v.d.B.); acschwulst@gmail.com (A.C.S.); bob.millar@up.ac.za (R.P.M.); claire.newton@up.ac.za (C.L.N.); 2 Department of Physiology, Faculty of Health Sciences, University of Pretoria, Private Bag X323, Pretoria 0031, South Africa; 3 Division of Endocrinology, Diabetes and Hypertension, Brigham and Women’s Hospital, Harvard Medical School, Boston, MA 02115, USA; songyb8686@gmail.com (Y.B.S.); ukaiser@bwh.harvard.edu (U.B.K.); 4 Division of Research Center, Scripps Korea Antibody Institute, Chuncheon 24341, Korea; 5 Department of Anatomy and Physiology, Faculty of Veterinary Sciences, University of Pretoria, Private Bag X04, Pretoria 0110, South Africa; 6 Department of Immunology, Faculty of Health Sciences, University of Pretoria, Private Bag X323, Pretoria 0031, South Africa; 7 Department of Integrative Biomedical Sciences, Institute of Infectious Diseases and Molecular Medicine, University of Cape Town, Observatory 7925, South Africa; 8 Deanery of Biomedical Sciences, University of Edinburgh, Edinburgh EH8 9JZ, UK; 9 School of Medicine, Medical and Biological Sciences Building, University of St Andrews, St Andrews KY16 9TF, UK

**Keywords:** neurokinin 3 receptor, pharmacological chaperone, G protein-coupled receptor, NKB

## Abstract

G protein-coupled receptors (GPCRs) facilitate the majority of signal transductions across cell membranes in humans, with numerous diseases attributed to inactivating GPCR mutations. Many of these mutations result in misfolding during nascent receptor synthesis in the endoplasmic reticulum (ER), resulting in intracellular retention and degradation. Pharmacological chaperones (PCs) are cell-permeant small molecules that can interact with misfolded receptors in the ER and stabilise/rescue their folding to promote ER exit and trafficking to the cell membrane. The neurokinin 3 receptor (NK3R) plays a pivotal role in the hypothalamic–pituitary–gonadal reproductive axis. We sought to determine whether NK3R missense mutations result in a loss of cell surface receptor expression and, if so, whether a cell-permeant small molecule NK3R antagonist could be repurposed as a PC to restore function to these mutants. Quantitation of cell surface expression levels of seven mutant NK3Rs identified in hypogonadal patients indicated that five had severely impaired cell surface expression. A small molecule NK3R antagonist, M8, increased cell surface expression in four of these five and resulted in post-translational receptor processing in a manner analogous to the wild type. Importantly, there was a significant improvement in receptor activation in response to neurokinin B (NKB) for all four receptors following their rescue with M8. This demonstrates that M8 may have potential for therapeutic development in the treatment of hypogonadal patients harbouring NK3R mutations. The repurposing of existing small molecule GPCR modulators as PCs represents a novel and therapeutically viable option for the treatment of disorders attributed to mutations in GPCRs that cause intracellular retention.

## 1. Introduction

G protein-coupled receptors (GPCRs) are heptahelical transmembrane receptors responsible for the majority of signal transductions across eukaryotic cell membranes. Given their physiological importance, many disorders are attributed to impaired GPCR signalling [1]. Loss-of-function mutations in GPCRs can render the receptors inactive by disrupting ligand binding or signal transduction/coupling to downstream effectors, but recent research endeavours have established that non-synonymous point mutations in GPCRs frequently result in receptor misfolding and subsequent reduced cell surface expression [2,3].

Endoplasmic reticulum (ER)-resident chaperones (collectively referred to as the ER quality control system (QCS)) can detect these misfolded mutants through various molecular and structural cues (including unpaired cysteines, exposed hydrophobic residues, and unprocessed N-glycans), promoting ER retention and ER chaperone-mediated refolding. Ultimately, should refolding fail, the mutant receptors are targeted for degradation via ER-associated degradation pathways (ERAD) [4,5]. This results in a reduced proportion of receptors entering the secretory pathway, with a concomitant decrease in cell surface-localised receptors.

To date, many disorders have been attributed to intracellular retention/impaired cell surface expression of GPCRs, including nephrogenic diabetes insipidus, retinitis pigmentosa, infertility, and obesity [6], with limited treatment options. However, retained receptors often preserve a degree of ligand binding and signalling competency [3], which raises the possibility that re-establishment of cell surface expression could subsequently restore receptor function. Chemical chaperones, such as 4-phenylbutiric acid, glycerol, and dimethyl sulfoxide, have been shown to non-selectively stabilise misfolded proteins, allowing them to escape the ER QCS [7,8]. More recently, hydrophobic cell-permeant small molecule ligand analogues have been identified that rescue cell surface expression and thereby the function of specific misfolded proteins, including GPCRs harbouring inactivating mutations that result in a failure to traffic to the cell membrane [3,9]. These selective molecules are referred to as pharmacological chaperones (PCs), or pharmacoperones, and represent a possible paradigm shift in the treatment of disorders attributed to GPCR intracellular retention. PCs are predicted to enter the ER and interact with nascent mutant misfolded proteins, promoting or “rescuing” their cell surface delivery. While the exact mechanisms behind PC rescue have yet to be definitively established, they are hypothesised to stabilise mutant protein structure through the formation of intramolecular interactions such that the proteins are no longer recognised as misfolded and can enter the secretory pathway [3,9]. In addition to rescuing cell surface expression of misfolded mutant GPCRs, PCs have also been demonstrated to increase the cell surface delivery of wild-type receptors, presumably by facilitating or enhancing their normal folding within the ER [10,11].

The study of PCs for pathogenic GPCR mutations remains an emergent field, but a number of studies have demonstrated their potential therapeutic viability. Improvements in disease phenotypes, including decreased urine production in diabetes insipidus patients harbouring vasopressin V_2_ receptor mutations, and restoration of testicular function in a mouse model of gonadotropin-releasing hormone (GnRH) receptor (GnRHR) retention [12,13], have been observed following PC treatment. A number of in vitro studies exploring the use of PCs to rescue cell surface expression of a range of misfolded GPCR mutants complement these in vivo studies and demonstrate the potential to develop this approach for the treatment of disorders attributed to GPCR retention [3].

Reproduction in vertebrates is driven by hypothalamic neuropeptide secretion. The neuropeptides regulate the activity of the hypothalamic–pituitary–gonadal (HPG) axis, and mediate their effects through GPCRs. Inactivating mutations in a number of these GPCRs have been shown to result in intracellular retention and infertility, including the LH receptor (LHCGR/LHR), FSH receptor (FSHR), and GnRHR, with all three being amenable to pharmacological chaperone rescue and restoration of signalling [13,14,15]. Neurokinin 3 receptor (NK3R, also known as TACR3) is the cognate GPCR for the neuropeptide neurokinin B (NKB) and represents a critical regulatory node of the HPG axis. Inactivating mutations in NK3R also result in reproductive dysfunction but in some cases the effect of these mutations on receptor trafficking/cell surface expression remains unknown [16,17,18,19,20,21].

The major physiological role ascribed to NK3R within the HPG axis is promoting the pulsatile release of the neuropeptide kisspeptin in a sex steroid-dependent manner from the hypothalamus [22,23,24,25]. However, it is a pleiotropic gene associated with a number of (patho)physiological processes, including hot flushing in menopausal women [26], mood disorders, chronic pain, and neurodegenerative disorders [27]. As a result, efforts have been made to develop NKB analogues (both peptide and non-peptide small molecule compounds) for potential therapeutic use. Development of non-peptide NKB analogues has generally been focussed on producing selective antagonists for application in the CNS field (e.g., anxiety, schizophrenia, pain) and for gastrointestinal tract pathologies such as irritable bowel syndrome, although trials to determine their therapeutic potential with respect to these applications have largely proved unsuccessful [22,23]. More recently, NK3R antagonists have been repurposed for the treatment of menopausal vasomotor symptoms (hot flushes) [20,21,25]. NK3R antagonists may also have applications in reproductive disorders requiring subtle modulation of gonadotropin and sex steroid levels, such as polycystic ovary syndrome, endometriosis, and benign prostatic hyperplasia [28].

**Figure 1:**
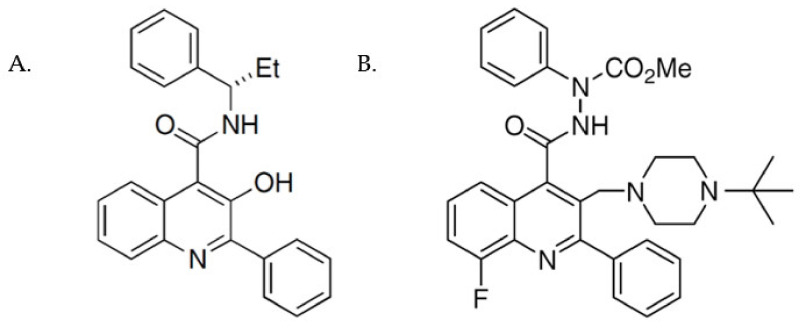
Chemical structures of NK3R antagonists. Chemical structures of the phenylquinoline-based NK3R antagonists talnetant (**A**) and M8 (**B**).

The non-peptide NK3R-targeting compounds fall into two main structural classes: dichlorophenylalkylpiperidines and quinolones. Talnetant (SB-223412) (Figure 1) was one of the first compounds developed within the latter class and the N’,2-diphenylquinoline-4-carbohydrazide-based compound, M8 (also known as 8m; Figure 1), was developed as part of a screen to identify compounds related to talnetant, with an improved pharmacokinetic profile and a reduction in cytochrome P450 induction [29]. M8 has been found to exhibit good receptor occupancy and selectivity [29].

In the current study we set out to determine: (1) whether mutations in the NK3R are associated with a failure to traffic to the cell surface, and (2) whether the cell surface expression and signalling of such intracellularly retained NK3R mutants could be restored by treatment with the NK3R antagonist M8. Quantitation of the cell surface expression of seven NK3R mutants revealed that five were severely intracellularly retained. M8 significantly increased cell surface expression of four of the intracellularly retained mutants, with a concomitant increase in signalling following stimulation with NKB.

## 2. Results

### 2.1. NK3R Missense Mutations Impair NKB-Stimulated Inositol Phosphate Generation

**Figure 2:**
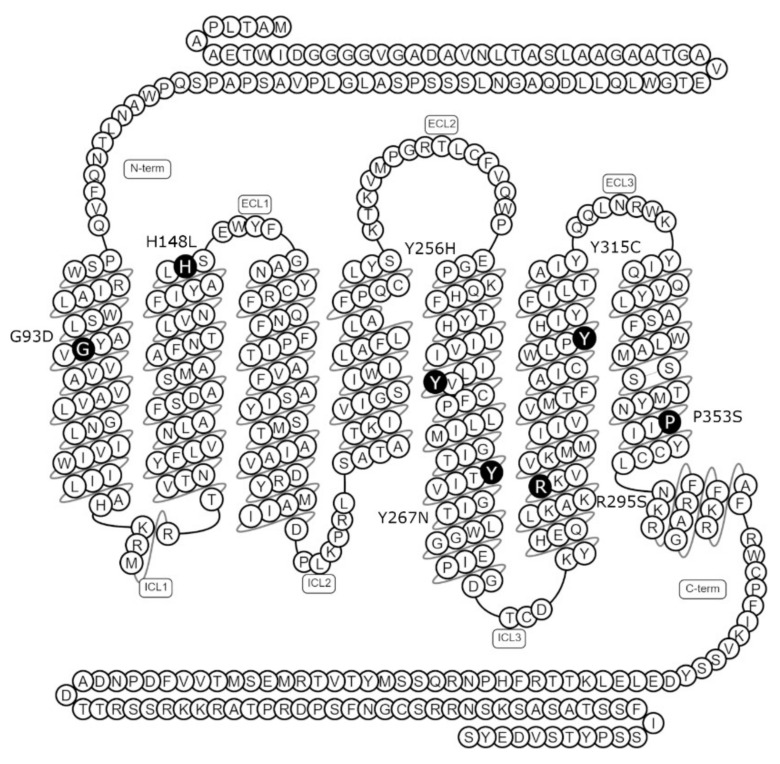
Schematic of the human NK3R amino acid sequence with positions of naturally occurring point mutations identified in patients with congenital hypogonadotropic hypogonadism. Positions of the non-synonymous point mutations considered in this study are indicated by black circles. N-term, N-terminus; ECL, extracellular loop; ICL, intracellular loop; C-term, C-terminus. Image generated using the GPCR database (gpcrdb.org, accessed on 22 March 2022) [30].

**Figure 3:**
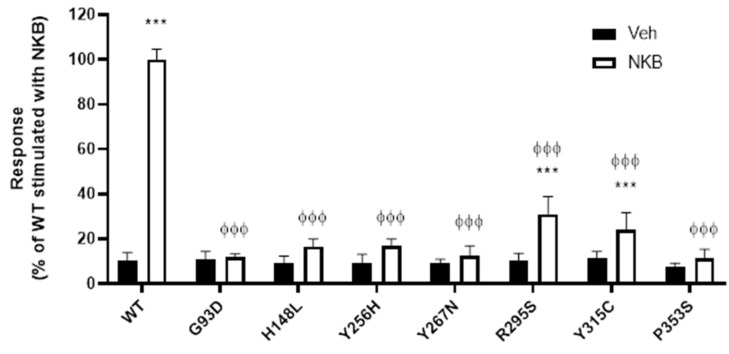
Signalling is impaired in NK3R mutants. Receptor signalling was measured using an inositol phosphate accumulation assay. HEK 293-T cells expressing WT or mutant NK3Rs were treated with 100 nM NKB (open bars) or vehicle (black bars) for 1 h. Data are expressed as percentage of WT stimulated with NKB (1766 ± 271 dpm), set to 100% and are presented as mean ± SEM from three independent experiments, in which each data point was performed in triplicate. *** *p* < 0.001, two-tailed Student’s *t*-test for comparison of NKB treatment with vehicle. ϕϕϕ *p* < 0.001, one-way ANOVA followed by Dunnett’s post hoc test for comparison of mutant receptors with WT NK3R signalling.

Seven missense mutations were selected from the literature for investigation. All seven mutations were identified in patients with congenital hypogonadotropic hypogonadism (CHH) (Figure 2). To confirm loss of function of these mutant receptors, their ability to respond to NKB stimulation was determined. As NK3R is a Gα_q_-coupled GPCR, measurement of inositol phosphate generation is an appropriate measure of receptor activity. Thus, cells expressing WT or mutant NK3Rs were stimulated with 100 nM NKB or vehicle and an inositol phosphate accumulation assay was utilised to quantify the resultant signalling responses. Basal activities of the mutant receptors were not significantly different from the basal activity of the WT receptor, but all of the mutant receptors had significantly reduced responses to NKB stimulation compared to the WT receptor response. Indeed, only mutants R295S and Y315C displayed any significant increase in NKB-induced signalling over basal activity and the responses elicited were only 31% and 24% of the WT response, respectively (Figure 3).

### 2.2. NK3R Missense Mutations Have Variable Effects on Receptor Cell Surface Expression

**Figure 4:**
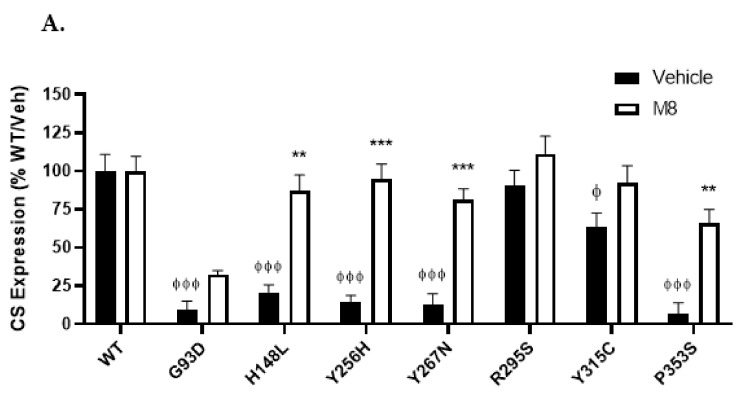

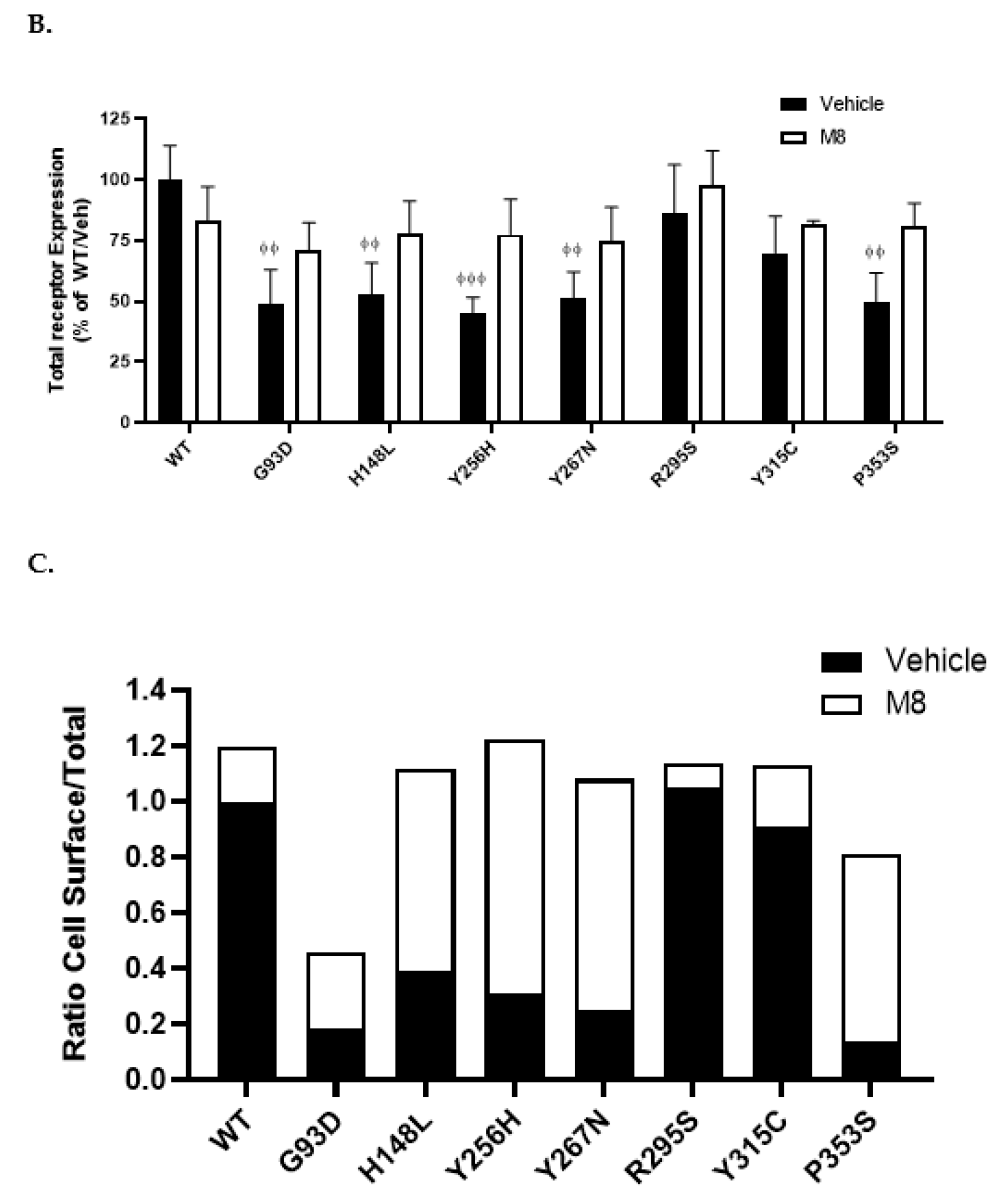
Cell surface expression of intracellularly retained mutant NK3Rs can be restored by M8 treatment. WT or mutant NK3R (**A**) cell surface (CS) (intact cells), or (**B**) total (permeabilised cells) receptor expression in transiently transfected HEK 293-T cells was examined in the presence or absence of 1 µM M8. Data are expressed as percentage of WT NK3R expression (untreated) following normalisation and background subtraction (vector transfected cells) and are presented as mean ± SEM from three independent experiments, in which each data point was performed in triplicate. ϕ *p* < 0.05, ϕϕ *p* < 0.01, ϕϕϕ *p* < 0.001, one-way ANOVA followed by Dunnett’s post hoc test for comparison of untreated mutant receptors with untreated WT NK3R. ** *p* < 0.01, *** *p* < 0.001, two-tailed *t*-test for comparison of vehicle and M8 treatment. (**C**) Mean data for receptor ELISAs were plotted as ratios of cell surface-to-total receptor expression, in the presence and absence of M8.

To establish whether the loss/reduction in NKB-induced signalling was due to reduced cell surface expression of the mutants, an enzyme-linked immunosorbent assay (ELISA) was utilised to quantitatively determine plasma membrane expression levels of the WT and mutant receptors. Six of the seven mutants exhibited a statistically significant reduction in cell surface expression when compared to WT (Figure 4A, black bars), with five of these resulting in a severe reduction in cell surface expression to <25% of WT levels (G93D, H148L, Y256H, Y267N, and P353S). Mutant R295S had cell membrane expression similar to WT, and Y315C had modestly (but significantly) reduced cell surface expression (64% of WT). These data were corroborated by a cell surface biotinylation assay, with only mutants H148L, R295S, and Y315C having detectable cell surface expression (Supplementary Figure S1, vehicle columns).

The reason for the significantly reduced NKB-induced signalling observed for the R295S and Y315C mutants, despite the fairly robust cell surface expression levels, was revealed upon radioligand-binding assays with ^125^I-labelled NKB. No NKB binding was measured in cells expressing mutant Y315C, indicating disrupted hormone binding (Supplementary Figure S2). While there was some reduction in NKB binding to cells expressing R295S mutant receptors, this was not sufficient to account for the severely reduced signalling, suggesting a deficit in signal transduction.

To determine whether the reduced cell membrane expression observed for the majority of the mutants was a result of an overall reduction in receptor expression (i.e., reduced de novo synthesis or increased receptor turnover/degradation, rather than decreased trafficking to the cell surface), the receptor ELISA assay was employed with permeabilised cells to allow measurement of both cell surface and intracellular receptor expression (referred to as ‘total’ receptor expression). All five mutations with severely reduced cell surface expression (G93D, H148L, Y256H, Y267N, and P353S) also had statistically significant reductions in total receptor expression (Figure 4B, black bars). Similar to the cell surface expression, mutant Y315C had a slight (although not statistically significant) reduction in total expression, while mutant R295S had a total expression not different from the WT receptor. Plotting the ratio of cell surface/total expression suggests that a combination of intracellular retention and reduced expression (via increased turnover or reduced synthesis) were responsible for the loss of G93D, H148L, Y256H, Y267N, and P353S mutant receptors at the cell surface (Figure 4C).

### 2.3. Cell Surface Expression of NK3R Mutants Is Rescued by the NK3R Antagonist, M8

As the majority (five out of seven) of the mutant receptors had severely impaired cell surface expression, the small molecule non-peptide NK3R antagonist, M8, was tested for PC activity, using the ELISA assay to monitor cell surface receptor expression following M8 treatment. Initial dose–response analyses with mutant Y256H determined that a concentration of 1 µM M8 was sufficient to elicit maximal rescue of this mutant receptor to the cell surface (Supplementary Figure S3). This concentration of M8 was therefore subsequently used to examine the rescue of cell surface expression of all the mutant receptors (Figure 4A, white bars). Following M8 treatment, four of the mutants showed a significant increase in cell surface expression to levels similar to that of the WT NK3R (H148L: 87%, Y256H: 95%, Y267N: 81%, and P353S: 66%; Cohen’s *d* effect size >1.69 when comparing vehicle to M8 treatment). The remaining three mutants (G93D, R295S, and Y315C) all had increased cell surface expression levels in response to M8 treatment, but these increases were not statistically significant. WT NK3R cell surface expression was unaffected by M8 treatment. Interestingly, talnetant, which shares the same core scaffold as M8 (Figure 1), was also able to increase cell surface expression of the H148L, Y256H, Y267N, and P353S mutants (Supplementary Figure S4), although these increases were less robust than those induced by M8 treatment. Cell surface biotinylation assays corroborated the findings of the ELISA assay and confirmed the increase in cell surface expression of these four mutant receptors upon M8 treatment (Supplementary Figure S1, M8 columns).

To determine whether the increased cell surface expression of these mutant receptors was due to M8 PC activity (i.e., promotion of cell surface trafficking of the mutant receptors) or an increase in total receptor expression in the cells, the same assay was employed using permeabilised cells (Figure 4B, white bars). In response to M8 treatment, all of the mutants (but not the WT receptor) underwent increases in total receptor expression, although none of these increases was statistically significant. The increased cell surface expression of mutant receptors H148L, Y256H, Y267N, and P353S therefore appears to be due to bona fide M8 PC activity.

To confirm the results of the receptor ELISAs, confocal microscopy was employed to visualise the cell surface rescue of the mutant receptors by M8. HEK 293-T cells were transfected with HA-tagged WT NK3R or Y267N NK3R. The cells were treated with either vehicle or 1 µM M8 before being probed with anti-HA primary antibody followed by a fluorescently labelled secondary antibody. In the absence of M8, WT NK3R localised mostly to the plasma membrane, while Y267N NK3R had observable intracellular distribution. M8 treatment resulted in a visible redistribution of Y267N to the cell surface (Supplementary Figure S5).

### 2.4. Post-Translational Modification of WT and Mutant NK3R

The receptor ELISA and confocal data demonstrate that M8 treatment restores cell surface delivery of the mutant NK3Rs. Nascent GPCR proteins are trafficked via the secretory pathway from the ER, through the ER–Golgi intermediate compartment (ERGIC) and Golgi apparatus en route to the cell surface. GPCRs can be extensively post-translationally modified during this process, with modifications such as glycosylation being important for cell surface delivery. As the NK3R contains three putative N-linked glycosylation consensus sequences (N-X-S/T, where X ≠ P), receptor glycosylation status was examined as a proxy for confirming whether the mutant receptors were entering the secretory pathway following M8 treatment.

**Figure 5:**
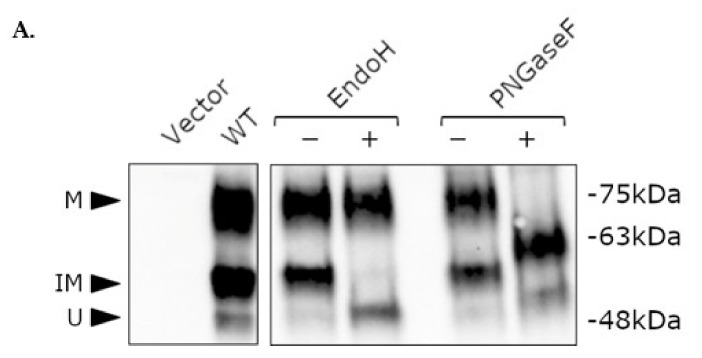

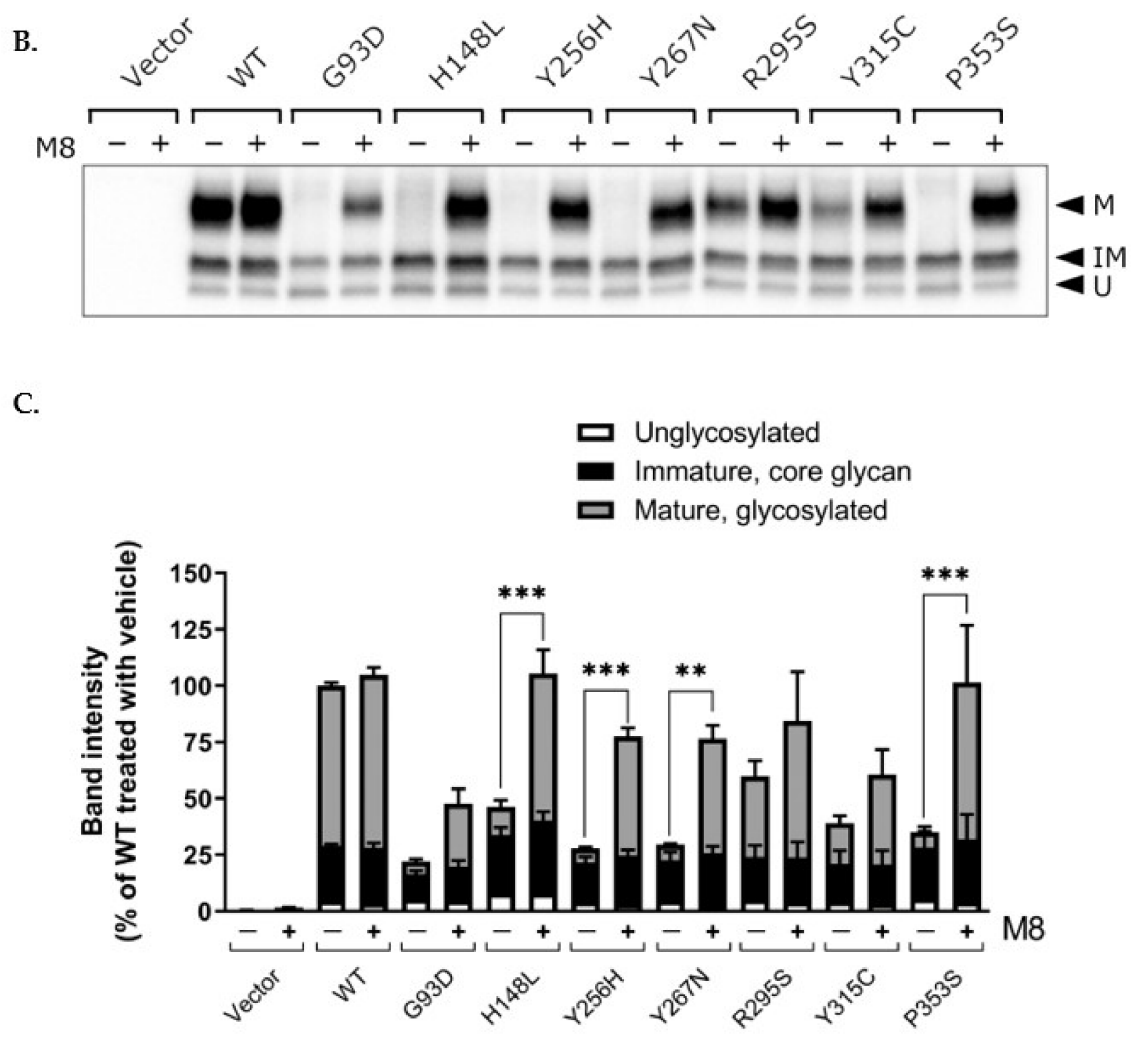
Mutant NK3Rs treated with M8 are post-translationally modified. (**A**) HEK 293-T cells transfected with empty vector or HA-tagged wild-type (WT) NK3Rs were lysed and subjected to Western blotting with an anti-HA antibody. Cell lysates were either untreated (left-hand panel) or were treated in the absence ((−), mock-treated) or presence ((+), enzyme-treated) of endoglycosidase H (EndoH) or peptide:N-glycosidase F (PNGaseF) enzymes to remove oligosaccharides. (**B**) HEK 293-T cells transfected with empty vector or HA-tagged WT or mutant NK3Rs were treated with 1 µM M8 (+) or vehicle (−), lysed, and subjected to Western blotting with an anti-HA antibody. Mature (M), immature (IM), and unglycosylated (U) receptors are indicated by black arrows. Image is representative of three independent experiments with similar results. (**C**) Densitometry of mature (M), immature (IM), and unglycosylated (U) receptor signals from (B) was performed using ImageLab 6.1.0 (BioRad Laboratories Inc., Hercules, CA, USA). Data are expressed as percentage of WT (treated with vehicle) and are presented as mean ± SEM from three independent experiments. ** *p* < 0.01 and *** *p* < 0.001, Student’s *t*-test for comparison of intensity of mature (M) band between M8 and vehicle treated cells.

Western blotting of HA-tagged WT NK3Rs transiently expressed in HEK 293-T cells revealed the presence of three specific bands (Figure 5A, left panel). The lowest band was the least intense and corresponded to the predicted molecular weight (MW) of unglycosylated NK3Rs of approximately 52 kDa. The two higher MW bands (approximately 60 and 75 kDa) were thought to represent post-translationally modified (glycosylated) NK3Rs. An enzymatic deglycosylation method was used to establish whether these higher MW species were indeed a result of N-linked glycosylation of the receptor. Endoglycosidase H (EndoH) cleaves high mannose and hybrid oligosaccharides from N-linked glycoproteins, thereby removing the core glycan from glycoproteins, and peptide-N-glycosidase F (PNGaseF) cleaves between the innermost N-acetylglucosamine and asparagine residues of high mannose, hybrid, and complex oligosaccharides, effectively removing all N-linked sugars from glycoproteins. When treated with these enzymes, mobility shifts for WT NK3Rs were detected (Figure 5A, right panel). EndoH treatment resulted in a shift of the intermediate 60 kDa band to approximately 52 kDa, reflecting removal of the core glycan, but had no effect on the higher 75 kDa band. PNGaseF treatment resulted in a shift of both the high and intermediate bands to lower molecular weights. These results suggest that NK3R is subjected to N-linked glycosylation, with three distinct molecular weight forms, representing unglycosylated (low MW), immature core glycan-containing (intermediate MW), and mature glycosylated receptor (high MW). In the case of WT NK3R, the mature receptor (high MW) is the predominant form at the cell surface, as indicated by cell surface biotinylation assays, in which the 75 kDa band was detected (Supplementary Figure S1).

The presence of these different receptor forms in the presence/absence of M8 treatment was then examined for the different mutant receptors (Figure 5B). For the five mutant receptors with severely impaired cell surface expression (G93D, H148L, Y256H, Y267N, and P353S), there was no detectable mature receptor in the absence of M8, with only bands corresponding to unglycosylated and immature glycosylated receptors present (Figure 5B). For mutants R295S and Y315C, which had unimpaired or moderately impaired cell surface expression respectively, all three MW bands were detected (Figure 5B). Following treatment with 1 µM M8, there was an increase in the number of high MW NK3Rs detected for all of the mutant receptors (Figure 5B,C). For the four mutants with significantly increased cell surface expression in the receptor ELISA (H148L, Y256H, Y267N, and P353S), these increases in mature modified receptors were statistically significant, and the signal for mature receptors following rescue was not statistically different from the mature WT receptor signal (H148L, 106%; Y256H, 76%; Y267N, 75%; and P353S, 95%). For all of the receptors tested, there were no significant changes in the number of unglycosylated or immature receptor forms (Figure 5B,C).

### 2.5. WT and Mutant NK3R Mutants Rescued by M8 Are Functional

The data presented thus far indicate that, in response to treatment with M8, there was a significant increase in cell surface expression of mutants H148L, Y256H, Y267N, and P353S, concomitant with an increase in mature, post-translationally modified receptors. To establish whether these ‘rescued’ mutant receptors were functional once at the cell surface, we examined their ability to bind hormones and respond to NKB stimulation.

M8 is thought to be an orthosteric antagonist. While many antagonists have been identified as effective PCs [3], displacement of the antagonist after cell surface rescue is a prerequisite for functional rescue. This can be challenging, as antagonist affinity positively correlates with ‘rescue potency’ [31,32,33]. To ensure sufficient antagonist displacement, such that NKB binding and NKB-induced receptor activation were not impaired by the presence of the antagonist, a wash-out procedure was employed prior to stimulation with NKB. The effectiveness of the washout procedure was determined by treating HEK 293-T cells expressing WT NK3R with 1 µM M8, washing to remove M8, and then stimulating with 100 nM NKB. There was only a small reduction in signalling compared to cells not treated with M8 (Supplementary Figure S6A), indicating that sufficient antagonist was removed to allow NKB-mediated stimulation of inositol phosphate accumulation. To confirm that ‘rescued’ mutant NK3Rs remained at the cell surface following wash-out of M8, a receptor ELISA was performed which indicated that the washout procedure did not appear to result in a substantial loss of cell surface expression of mutant receptors (Supplementary Figure S6B).

**Figure 6:**
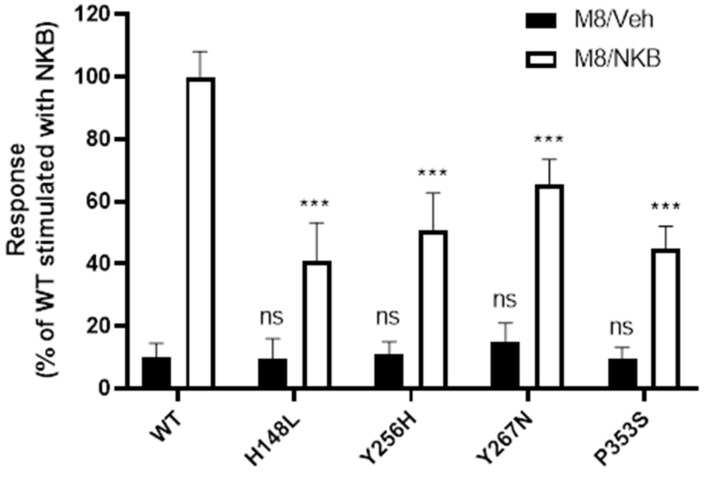
M8-mediated trafficking of intracellularly located NK3R mutants to the cell membrane restores NKB-mediated stimulation of inositol phosphate accumulation. Receptor signalling was measured using an inositol phosphate accumulation assay. HEK 293-T cells expressing WT NK3Rs or mutant NK3Rs were pre-treated with 1 µM M8 for 24 h. Cells were then washed for 3 h before treatment for 1 h with vehicle (black bars) or 100 nM NKB (white bars). Data are expressed as percentage of WT NK3Rs stimulated with NKB (1582 ± 239 dpm), set to 100% and are presented as mean ± SEM from three independent experiments, in which each data point was performed in triplicate. *** *p* < 0.001, Student’s *t*-test for comparison of NKB treatment with vehicle. ns *p* > 0.05, one-way ANOVA followed by Dunnett’s post hoc test for comparison of basal signalling of mutant receptors with basal signalling of WT NK3Rs.

Signalling competence of the rescued mutants in response to the endogenous ligand, NKB, following M8 treatment was then examined. Cells expressing WT or severely retained mutant NK3Rs were pre-incubated with 1 µM M8, washed, and treated with vehicle or 100 nM NKB. Following M8 pre-incubation, basal activities of the mutant receptors were not significantly different from the WT receptor (Figure 6, black bars). Strikingly, all four mutants pre-treated with M8 and stimulated with NKB showed significant increases in NKB-induced inositol phosphate generation over unstimulated mutant receptors (H148L to 41%, Y256H to 51%, Y267N to 65%, and P353S to 45% of the NKB-stimulated WT NK3R response; Cohen’s *d* effect size >1.63 when comparing vehicle to NKB treatment) (Figure 6).

## 3. Discussion

The NK3R plays an important role in the regulation of the HPG axis and has also been associated with a number of other (patho)physiological processes. Thus, dysfunction of NK3R signalling through loss-of-function mutations has implications for human health and fertility [34]. Evidence from our group, and others, has suggested that many GPCR point mutations result in impaired cell surface receptor expression and are therefore candidates for PC therapy [3,35]. To date, most GPCR PCs have been identified through the repurposing of existing small molecule modulators of receptor activity [36]. There are many NK3R small molecule orally bioavailable antagonists currently in various stages of development, some of which have proven efficacious in the treatment of vasomotor symptoms [37]. These molecules were well tolerated, with side effects largely limited to mild gastrointestinal disorders and small transient rises in liver transaminases [37], suggesting that the repurposing of NK3R antagonists as pharmacological chaperones could be therapeutically viable. In this paper, we have quantitatively described the expression and signalling ability of seven non-synonymous point mutations previously designated as deleterious to NK3R function [16,17,18,19,20,21,38], and we demonstrate that the majority indeed have impaired cell surface expression. Moreover, we show that an existing non-peptide small molecule NK3R antagonist, M8, can rescue cell surface expression and increase the NKB-mediated signalling response of mutant receptors with impaired cell surface expression.

### 3.1. NK3R Mutations G93D, H148L, Y256H, Y267N, and P353S Result in Severely Impaired Cell Surface Expression

When cell surface expression was examined, six out of seven (86%) of mutants had reduced expression at the cell surface, with five (G93D, H148L, Y256H, Y267N, and P353S) exhibiting severely impaired cell surface expression (Figure 4A). This proportion is in accordance with other publications characterising inactivating point mutations in GPCRs, including the V_2_ vasopressin receptor [39], the luteinising hormone receptor [40], and the follicle-stimulating hormone receptor [15], in which approximately 90% of examined mutations resulted in intracellular retention/loss of plasma membrane receptor expression, reinforcing the notion that intracellular retention is the major mode of inactivation of GPCRs harbouring deleterious point mutations [2].

For the five mutants with severely impaired cell surface expression, there was a concomitant reduction in total cellular expression levels to approximately 50% of WT levels, but examination of the ratio of cell surface-to-total expression indicated that reduced cell surface trafficking (rather than decreased total expression of the receptors) is the major reason for the reduced cell surface expression of these mutants. It is likely that the reduced total expression of these receptors is due to activation of ERAD pathways as a result of terminal misfolding, leading to protein degradation, but confirming the mechanisms underlying the reduced total expression was beyond the scope of this study. Loss of cell surface expression for these five mutants (G93D, H148L, Y256H, Y267N, and P353S) is therefore likely to be the primary cause of their loss of function/reduced response to NKB-stimulation and therefore the reproductive phenotypes that were observed in the patients in which they were identified [16,17,18,19].

The homozygous G93D mutation was identified in two Turkish siblings with normosmic hypogonadotropic hypogonadism (HH). This residue is located in transmembrane (TM) helix 1 (Figure 2) and only one non-olfactory class A GPCR contains an aspartic acid residue in this position (position 1.40, Ballesteros–Weinstein numbering system [41]). The introduction of a charged (acidic) residue into this hydrophobic transmembrane domain is therefore likely to be disruptive to receptor conformation/folding.

In contrast, the H148L mutation identified in three Kurdish siblings [17] is located at the interface of TM helix 2 and extracellular loop (ECL) 1 (Figure 2), in close proximity to two asparagine residues predicted to be of importance for NKB binding [42]. Functional studies established that H148L results in a loss of activity [17], with our results confirming that this is due to severe intracellular retention of the mutant. Replacement of a large charged (basic) residue with a small non-polar leucine at this position possibly disrupts intermolecular interactions important for receptor conformation.

Mutation Y256H is located in TM helix 5 (Figure 2). In this location (position 5.47), only phenylalanine is more frequently found in the non-olfactory class A GPCRs, indicating that the presence of a hydrophobic aromatic side chain is important in this position. Again, like mutation G93D, introduction of a charged (in this case, basic) residue into a hydrophobic transmembrane domain is likely to be disruptive. The finding that this mutation severely impairs cell surface expression is in agreement with a previous study characterising the effects of this mutation [43]. Interestingly, the same authors describe a loss of total cellular expression (determined by Western blotting) [43], yet we only observed a reduction of total expression of approximately 50%, although differences in assay sensitivity may underline this discrepancy.

Mutation Y267N in TM helix 5 (Figure 2) was identified in a proband in a compound heterozygous state along with an NK3R nonsense mutation (Y267N/W275X). The proband was diagnosed with normosmic HH presenting with low plasma testosterone (0.4 ng/ mL) and serum LH (0.65 IU/L) [19]. Mutation Y267N might also be expected to be deleterious to receptor activity, given that this location (position 5.58) is highly conserved, being occupied by a tyrosine in 73% of non-olfactory class A GPCRs, and in a subgroup of rhodopsin family GPCRs has been identified as a residue of importance for receptor activation through interaction with the NPxxY molecular switch in TM helix 7. In agreement with the present study, the authors that first identified and characterised this mutation also described a loss of cell surface expression for this mutant receptor and hypothesised that this was due to misfolding as a result of the introduction of a polar side chain into the local hydrophobic environment.

The P353 residue (position 7.50) represents one of the most conserved residues in non-olfactory class A GPCRs, with 93% of receptors containing a proline in this position. As with Y267 and Y315, this proline falls within a well-characterised rhodopsin family molecular switch, in this case NPxxY, which facilitates the rotational movement of TM 7 during receptor activation. Proline residues cause ‘kinking’ of alpha helixes, which is often important for receptor activity/intra-molecular interactions, and their replacement is likely to have deleterious effects on receptor conformation as well as function. Topaloglu et al. described a loss of signalling as a result of the P353S mutation [16], with our data implying that this is most likely due to misfolding and loss of cell surface expression.

The severely impaired cell surface expression of mutants G93D, H148L, Y256H, Y267N, and P353S correlated with little or no mature glycosylated receptors. Glycosylation is the most common post-translational modification of GPCRs, affecting many aspects of GPCR biology. Indeed, the closely related NK1R has two N-linked glycosylation sites, and site-directed mutagenesis of these has been shown to result in a 50% reduction in binding of its cognate ligand, substance P, and the promotion of a more rapid internalization of the receptor following stimulation [44]. Importantly, glycosylation is also a rate-limiting step governing ER exit and entry into the secretory pathway [45,46,47,48]. The NK3R contains three consensus sites for N-linked glycosylation and multiple serines and threonines (residues modified by O-linked glycosylation). N-linked glycosylation occurs co-translationally within the ER, whereby a core N-glycan (N-acetylglucosamine, GlcNAc) is added to an asparagine situated within the consensus sequence (Asn-X-Ser/Thr (X ≠ Pro)), followed by further modification and recognition by lectin-like ER resident folding chaperones such as calnexin and calreticulin, promoting ER exit. Further processing of these glycans is performed within the Golgi as the receptor is trafficked to the cell surface via the secretory pathway. Western blotting analyses indicated that the NK3Rs expressed in HEK 293-T cells exist in three distinct forms of approximately 52, 60, and 75 kDa (Figure 5A), with the 52 kDa form corresponding to the predicted MW of (unglycosylated) NK3R. Enzymatic deglycosylation confirmed that the intermediate band represented NK3Rs modified by the addition of core N-glycans. Interestingly, the predicted MW of the core N-glycan is 2.4 kDa, implying that all three available asparagines within the N-terminus may be glycosylated. The presence of the highest MW band is suggestive of further post-translational modification (PTM) of NK3Rs following ER exit, and biotinylation assays confirmed that this form represents the ‘mature’ cell membrane-localised receptor (Supplementary Figure S1). Although the molecular weight of this receptor species was reduced by PNGaseF treatment, it was not decreased to that of the unglycosylated receptor (Figure 5A), suggesting that PTMs other than N-linked glycosylation are present on the mature receptors, or, alternatively, that the NK3R core GlcNAc is α(1–3) fucosylated, which prevents cleavage by PNGaseF. The observation that the severely impaired cell surface expression of the mutant receptors was due to a partial or complete absence of mature receptors (Figure 5B), despite the mutant receptors still being modified by the addition of the core N-glycans (indicated by the presence of the intermediate band), suggests that the nascent receptors were possibly being modified in the ER but that misfolding was preventing their exit and further PTM.

### 3.2. M8 Can Restore the Impaired Cell Surface Expression of H148L, Y256H, Y267H, and P353S Mutant NK3Rs

When the five mutants with severely impaired cell surface expression were treated with the small molecule antagonist, M8, an increase in receptor cell surface expression was observed, with these increases being significant for mutants H148L, Y256H, Y267N, and P353S (Figure 4A). That talnetant mirrored the effects of M8 on cell surface expression of these mutant receptors (Supplementary Figure S4) is unsurprising as they are closely related compounds (Figure 1) and therefore likely to interact with the receptors in a similar manner. Following M8 treatment, those mutants with increased cell surface expression also exhibited an increase in the presence of receptors in the mature (high MW) form (Figure 5B), indicating that trafficking and PTM were restored. The rescue of cell surface expression of these mutant receptors suggests that M8 binding directly or indirectly stabilizes TM helices 2, 5, and 7, in which these mutations are located, such that they are able to bypass the ER QCS and restore trafficking. However, structural modelling analyses would be required to confirm this.

Although M8 treatment induced a slight increase in cell surface expression of mutant G93D, this was minor and not statistically significant. Pertinently, it has previously been demonstrated that talnetant binding is sensitive to a laboratory-generated V95A mutant that is in close proximity to G93D [42], suggesting that disruption of this region of the receptor may also directly/indirectly affect binding of the closely related M8 and may explain the lack of M8-induced rescue of cell surface expression observed for this mutant receptor. Alternatively, it is possible that M8 does not interact with TM helix 1 or does so in a manner that is insufficient to stabilise conformational defects elicited by this mutation.

### 3.3. Increased Cell Surface Expression Restores Function to the Rescued Mutant Receptors

For the severely retained mutants that could be rescued by M8 treatment (H148L, Y256H, Y267N, and P353S), the increase in cell surface expression observed following M8 treatment translated into a concomitant increase in signalling response (Figure 6). Interestingly, this increase in signalling response was not proportional to the observed increases in cell surface expression.

It was somewhat surprising that mutations P353S and Y267N were (at least to a degree) signalling competent following rescue of cell surface expression by M8. The very highly conserved P353 (position 7.50) falls within a well-characterised rhodopsin family molecular switch, NPxxY. Upon receptor activation, TM 7 is predicted to rotate around this proline to bring together and facilitate hydrogen bonding between the tyrosine of the NPxxY (position 7.53) and residue Y267 (position 5.58) in TM 5. These data therefore suggest that the NK3Rs may utilise an alternative mechanism of activation or different intramolecular interactions to stabilise the active conformation.

For mutants H148L and Y256H, it is entirely plausible that the lack of concordance between cell surface rescue and the magnitude of signalling response following M8 treatment may be explained by additional defects relating to NKB binding and/or signal transduction induced by the presence of these mutations. Molecular modelling and mutagenesis studies suggest that NK3R is activated via insertion of the C-terminus of NKB into a hydrophobic binding pocket formed by TM helices 2, 3, 6, and 7 of the transmembrane bundle [42,49]. Indeed, mutation H148L is located in close proximity to two asparagine residues, N138 (position 2.57) and N142 (position 2.61), predicted to be of importance for NKB binding [42], and its location at the interface of ECL 1 and TM 2 places it in the region of the NKB binding site. Small molecule modulators of NK3R activity have been shown to utilise a pocket lying deep within the TM bundle that is distinct but overlaps with the NKB site [42]. Therefore, due to their distinct binding sites, small molecule ligands such as M8 may be able to interact with (and rescue cell surface expression of) mutant receptors, in which NKB binding interactions are disrupted. Although residue Y256, which is located in TM 5, lies deeper within the TM bundle, it is possible that the disruption of receptor conformation/intramolecular interactions induced by the replacement of tyrosine with histidine indirectly results in disruption of the NKB binding site (although presumably not the M8 binding site, as M8-induced rescue of cell surface expression is maintained).

### 3.4. Y315C and R295S Mutant NK3Rs Have Good to Moderate Cell Surface Expression but Impaired NKB Binding and/or Signalling

Two of the mutant receptors displayed modest (Y315C) or no (R295S) impairment in cell surface expression. Although mutant Y315C had cell surface expression at 64% of WT levels (Figure 4A), no NKB binding was measured (Supplementary Figure S2). This lack of binding correlates with a complete lack of NKB-induced signalling by this mutant receptor (Figure 3). These findings are in accordance with previously published data that revealed a severe loss of signalling and a reduction, but not ablation, of trafficking of this mutant receptor to the plasma membrane [43]. Y315, located in TM 6 (position 6.51), is relatively well conserved, with 66% of non-olfactory class A GPCRs containing a hydrophobic aromatic amino acid in this position (most commonly tyrosine). The residue falls within a region of TM 6 with high sequence conservation and is located towards the extracellular end of the helix. Molecular modelling has identified Y315 as a critical determinant of NKB binding and, with neighbouring residues C311 (position 6.47) and W312 (position 6.48), it forms part of the hydrophobic ligand binding pocket [42,49]. It is perhaps, therefore, unsurprising that loss of NKB binding was observed when the large aromatic tyrosine was replaced with cysteine at this position.

Mutation R295S, also located in TM 6, had unimpaired cell surface expression (Figure 4A) but the binding of NKB was reduced to 55% of WT levels (Supplementary Figure S2) and NKB-induced signalling to 31% of WT levels (Figure 3), suggestive of impairments in both NKB binding and signalling. Again, these data are in agreement with the data of Noel et al., who described a very slight decrease in cell surface expression, a modest reduction in NKB binding, and a large reduction in inositol phosphate accumulation upon NKB stimulation of this mutant receptor [43]. It is unclear how this mutation may affect NKB binding, but presumably replacement of the large charged (basic) arginine with a small uncharged serine disrupts the intermolecular interactions required for stabilising conformation of the NKB binding pocket. The location of R295 at the TM helix 6/intracellular loop (ICL) 3 boundary (position 6.31) may explain the loss of signalling by this mutant receptor. ICL 3 has been shown to be an important determinant of G protein binding and selectivity in class A GPCRs [50,51]. Despite the robust effects of this mutation on NKB binding and NKB-mediated signalling, R295S is not retained, suggesting that it is not recognised as misfolded by the ER QCS. This region is often characterised in crystal structures as disordered and proteolytically susceptible, implying that it is unstable, at least in the absence of interacting proteins [52], which might indicate tolerance to certain amino acid substitutions. Indeed, position 6.31 is not highly conserved among the non-olfactory class A GPCRs and this may account for the lack of effect of this mutation on receptor folding/cell surface expression.

## 4. Materials and Methods

### 4.1. Materials

Neurokinin B was purchased from EZBiolab (Carmel, IN, USA). M8 (also known as 8m [29]) and talnetant were kindly supplied by Dr Graeme Fraser (Ogeda SA, Brussels, Belgium) and Dr Mike Trower (NeRRe Therapeutics, Stevenage, UK), respectively. Primary mouse monoclonal anti-HA tag antibody (5B1D10; cat. no. 32-6700) was purchased from Thermo Fisher Scientific (Waltham, MA, USA) and Goat Anti-Mouse IgG (H + L)-HRP Conjugate (cat. no. 170-6516) was purchased from Bio-Rad Laboratories (Hercules, CA, USA). Endoglycosidase H (EndoH; P0702S) and peptide-N-glycosidase F (PNGaseF; P0704S) were purchased from New England Biolabs (Ipswich, MA, USA). All other general laboratory reagents were obtained from Sigma-Aldrich (St Louis, MO, USA) unless stated otherwise.

### 4.2. Site-Directed Mutagenesis

Plasmids encoding NK3R mutants G93D, H148L, Y256H, Y267N, and P353S were created by site-directed mutagenesis using a QuikChange site-directed mutagenesis kit (Agilent, Santa Clara, CA, USA) using the WT NK3R (WT HA-NK3R) as a template (NM_001059.3). (See Supplementary Table S1 for mutagenesis oligonucleotide sequences.) Mutations were verified by Sanger sequencing.

### 4.3. Cell Culture and Transfection

Human embryonic kidney cells stably expressing large T antigen (HEK 293-T; ATCC, Manassas, VA, USA, cat. no. CRL-3216) were cultured in Dulbecco’s Modified Eagle Medium (DMEM) containing GlutaMAX (Thermo Fisher Scientific, Waltham, MA, USA), supplemented with 10% (*v/v*) foetal calf serum (FCS) at 37 °C, 5% CO_2_, and 95% humidity. All assay plates were pre-treated with a 1:30 dilution of Matrigel Growth Factor Reduced Basement Membrane Matrix (Corning, Corning, NY, USA) to aid cell attachment. The plasmids encoding WT or mutant HA-NK3Rs were transiently transfected into HEK 293-T cells using XtremeGENE HP DNA transfection reagent (Sigma-Aldrich, St. Louis, MO, USA) at a 2:1 (XTG:DNA) ratio.

COS-7 (ATCC, cat. no. CRL-1651) cells were maintained in DMEM supplemented with 10% FCS and antibiotics. The plasmids encoding WT or mutant HA-NK3Rs were transfected into COS-7 cells using Lipofectamine2000, following the manufacturer’s instructions (Invitrogen, Waltham, MA, USA).

### 4.4. Inositol Phosphate Accumulation Assay

HEK 293-T cells were plated at 2 × 10^5^ cells per well in Matrigel-coated 12-well plates, and, 24 h post-seeding, the cells were transfected with WT HA-NK3R or mutant NK3Rs. Cells were treated with M8 (1 µM) or vehicle (0.1% DMSO) 24 h post-transfection and incubated for a further 24 h in media 199 (Thermo Fisher Scientific, Waltham, MA, USA) containing tritiated-myoinositol (0.5 µCi/mL) (PerkinElmer, Waltham, MA, USA) before being washed in buffer I (DMEM supplemented with 20 mM HEPES and 0.1% BSA) to ensure removal of M8 (one wash, incubation for 3 h in buffer I and then a final wash). Cells were then incubated with buffer I supplemented with 10 mM LiCl for 30 min at 37 °C before stimulation with 100 nM NKB or vehicle for 1 h at 37 °C. Cells were lysed in 10 mM formic acid and radioactive inositol phosphates isolated by ion-exchange chromatography before the addition of scintillation fluid (Optiphase HiSafe; PerkinElmer, Waltham, MA, USA) and measurement on a Packard Tri-carb 2100TR liquid scintillation analyser (PerkinElmer, Waltham, MA, USA). To normalise for inter-assay variability, the sum of all data values for each biological repeat was calculated and each data point within that biological repeat was divided by this sum to calculate a ratio. Data were then expressed as a percentage of the values measured for WT HA-NK3R transfected cells. Data were analysed using GraphPad Prism (Version 9) software (GraphPad Inc, San Diego, CA, USA) by Student’s *t*-test for comparison of NKB treatment with vehicle and one-way ANOVA followed by Dunnett’s post hoc test for comparison of mutant NK3R signalling with WT NK3R signalling.

### 4.5. Receptor ELISA

HEK 293-T cells were plated at a density of 0.8 × 10^5^ cells per well in Matrigel-coated 48-well plates. Twenty-four hours post-seeding, the cells were transfected with empty vector, WT HA-NK3R, or mutant NK3Rs, and, 24 h post-transfection, the cells were treated with 1 µM M8, 1 µM talnetant, or vehicle (0.1% DMSO) and incubated for a further 24 h. A receptor ELISA was performed on intact or permeabilised cells (to quantify cell surface or total receptor expression, respectively), as previously described [14], with the exception that a primary antibody targeting the N-terminal HA-epitope tag was used (final concentration 0.5 µg/mL). To normalise for inter-assay variability, the sum of all data values for each biological repeat was calculated and each data point within that biological repeat was divided by this sum to calculate a ratio. Data were then expressed as a percentage of the values measured for WT HA-NK3R transfected cells (treated with vehicle) after subtraction of non-specific signals (measured in cells transfected with empty vector). Data were analysed using GraphPad Prism (Version 9) software (GraphPad Inc., San Diego, CA, USA) by two-tailed Student’s *t*-test for comparison of vehicle treatment and M8 treatment or by one-way ANOVA followed by Dunnett’s post hoc test for comparison of mutant and WT receptors, with *p* < 0.05 considered significant.

### 4.6. Western Blotting and Deglycosylation Assay

HEK 293-T cells were plated at a density of 4 × 10^5^ cells per well in Matrigel-coated 6-well plates. Twenty-four hours post-seeding, cells were transfected with empty vector, WT HA-NK3R or mutant NK3Rs, and, 24 h post-transfection, the cells were lysed for 15 min at 4 °C in lysis buffer (10 mM Tris-HCl, 150 mM NaCl, 1% (*v/v*) IGEPAL Ca-630, pH 7.2), cleared by centrifugation (16,000 g, 10 min, 4 °C), and protein concentrations were determined by bicinchoninic acid (BCA) assay (Thermo Fisher Scientific, Waltham, MA, USA, cat. no. 23225).

Lysates prepared from cells expressing WT HA-NK3Rs or empty vectors were subjected to EndoH, PNGaseF, or mock treatments (New England Biolabs, Ipswich, MA, USA), as per the manufacturer’s instructions. The lysates (40 µg) were incubated for 30 min at room temperature in glycoprotein denaturation buffer (New England Biolabs, Ipswich, MA, USA) followed by incubation with 1000 U of enzyme for 1 h at 37 °C.

Cell lysates were incubated at room temperature for 1 h in protein-loading buffer (62.5 mM Tris-HCl (pH 6.8), 2% (*w/v*) SDS, 10% glycerol, 0.01% (*w/v*) bromophenol blue, 5% (*v/v*) beta-mercaptoethanol) before 15 µg was loaded onto 15-well 4–20% Mini-PROTEAN TGX Stain-Free Protein Gels (Bio-Rad Laboratories Hercules, CA, USA, cat. no. 4568096). The samples were electrophoresed and transferred to polyvinylidene difluoride (PVDF) membranes. The membranes were then blocked in blocking solution (5% (*w/v*) low-fat milk powder in phosphate-buffered saline (PBS)) for 1 h at room temperature and probed with 0.5 µg/mL monoclonal anti-HA primary antibody, prepared in blocking solution, overnight at 4 °C. The membranes were then washed with PBS containing 0.1% (*v/v*) Tween-20 (PBST) and probed for 1 h at room temperature with 0.4 µg/mL HRP-conjugated secondary antibody in blocking solution. The membranes were then washed in PBST and incubated with Clarity Western ECL Substrate (Bio-Rad Laboratories Hercules, CA, USA, cat. no. 1705061) then analysed on a ChemiDoc Imaging System (Bio-Rad, Hercules, CA, USA) using Image Lab software version 6.1.0 (Bio-Rad, Hercules, CA, USA). Densitometric quantification of signals was performed on a minimum of three independent experiments to generate data for statistical analysis. The stain-free acrylamide gels utilise trihalo compounds within the gel matrix that fluoresce when bound to proteins, allowing imaging prior to transfer of proteins to PVDF for determination of total protein present in each lane (see [53]). All data points were therefore normalised to the corresponding value for total protein in the appropriate lane and then calculated as a percentage of the WT receptors (vehicle-treated). Data were analysed using GraphPad Prism (Version 9) software (GraphPad Inc, San Diego, CA, USA) by two-tailed Student’s *t*-test for comparison of vehicle treatment and M8 treatment, or by one-way ANOVA followed by Dunnett’s post hoc test for comparison of mutant and WT receptors, with *p* < 0.05 considered significant.

## 5. Conclusions

Chemical chaperones have proven to be efficacious in the treatment of protein misfolding and protein aggregation disorders in animal models [7], but issues regarding target specificity and the high doses required have prevented their development as therapeutics. Moreover, as they are not specific, it is likely that a wide range of normal GPCRs will be non-specifically upregulated. The development of PCs that offer target specificity at pharmacologically relevant doses represents a paradigm shift in the way that we approach the treatment of disorders attributed to protein misfolding. GPCRs are an excellent example of a physiologically important family of proteins that would be amenable to PC treatment given that for many identified GPCR mutations, intracellular retention appears to be the major mode of receptor inactivation [2,40]. Here, we have demonstrated that missense mutations of the NK3R also predominantly result in loss of receptor cell surface expression, which can account for the non-functionality of the receptors and subsequent disease phenotypes. Promisingly, the cell surface expression of the majority of these mutant receptors could be restored to significant levels by treatment with a small molecule antagonist, M8. Despite the mutations also causing some impairment in hormone binding and/or signalling of the rescued mutant receptors, their functionality (i.e., responsiveness to NKB stimulation) was increased by M8 rescue. PC rescue of mutant NK3Rs, therefore, may represent a valid therapeutic option in the treatment of hypogonadism resulting from NK3R-inactivating point mutations which cause intracellular retention and poor cell surface expression. Although such patients could be treated with downstream hormones of the HPG axis to restore fertility, treatment with PCs would provide a more subtle and controlled therapeutic option, as the spatial and temporal activity of endogenous NKB hormone would be restored. Furthermore, NK3R is a pleiotropic gene and, while administration of reproductive hormones would potentially resolve reproductive axis pathologies, PC treatment would have the added benefit of restoring all functions ascribed to this receptor. While this study successfully establishes that M8 has PC activity, a limitation is that the mechanisms behind M8 rescue of intracellularly retained mutant NK3Rs remains elusive. Future studies to establish how M8 facilitates transmembrane helix stabilisation and the mechanisms behind PC-mediated ER escape would be both informative and important in the context of developing PCs therapeutically.

## Data Availability

The data presented in this study are available on request from the corresponding author (RCA).

## Supplementary Materials

The supporting information can be downloaded at: https://www.mdpi.com/article/10.3390/ijms23094587/s1.
