# Supplementary material for: Functional Rescue of Inactivating Mutations of the Human Neurokinin 3 Receptor Using Pharmacological Chaperones
Source: Int J Mol Sci. 2022 Apr 21;23(9):4587. doi: 10.3390/ijms23094587 (PMC9100388; doi:10.3390/ijms23094587)
Supplement: Supplementary file 1 [file ijms-23-04587-s001.zip › Supplementary Figures.pdf]

SUPPLEMENTAL FIGURES

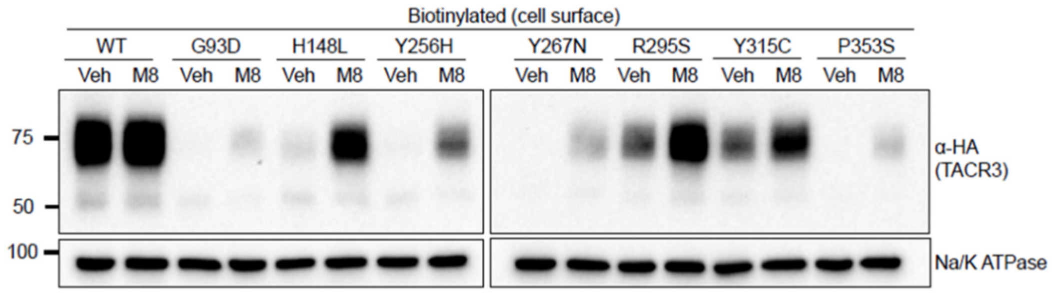

Figure S1: Cell surface expression of mutant NK3Rs is increased by M8. HEK 293-T cells transfected with WT or mutant NK3Rs were treated with vehicle (Veh) or 100 nM M8 for 24 h. Cell surface fractions were isolated and immunoblotted, and NK3Rs were detected with anti-HA antibody. Na/K ATPase was utilised as a control to confirm isolation of similar amounts of cell surface proteins among samples.

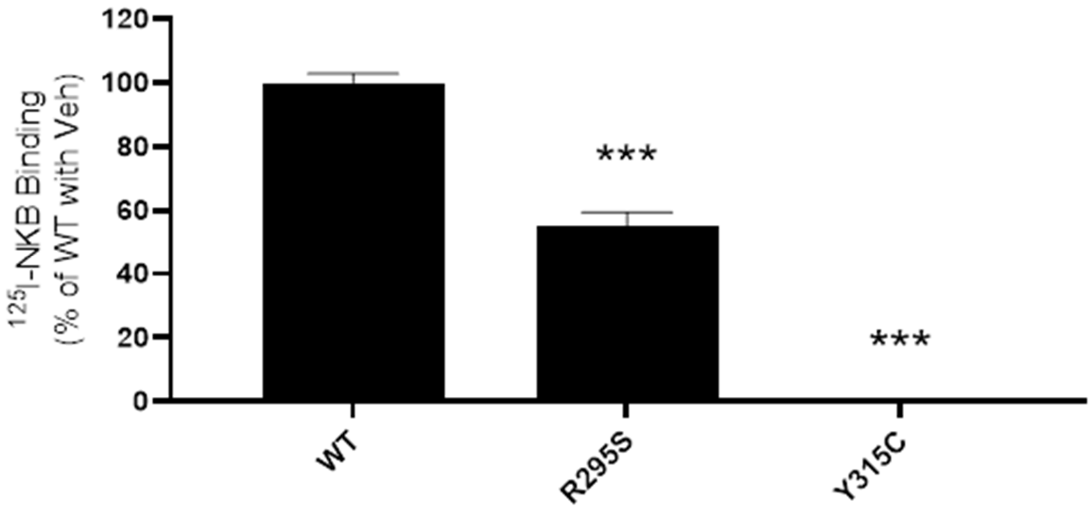

Figure S2: R295S and Y315C mutant NK3Rs have impaired hormone binding. Ability of mutants to bind NKB was measured by radioligand-binding. COS-7 cells were transfected with WT or mutant NK3Rs. Data are expressed as percentage of NKB binding to WT receptor following normalisation and background subtraction and are presented as mean ± SEM from three independent experiments, in which each data point was performed in triplicate. *** $p<0.001$, one-way ANOVA followed by Dunnett's post-hoc test for comparison of mutant NK3Rs with WT NK3R.

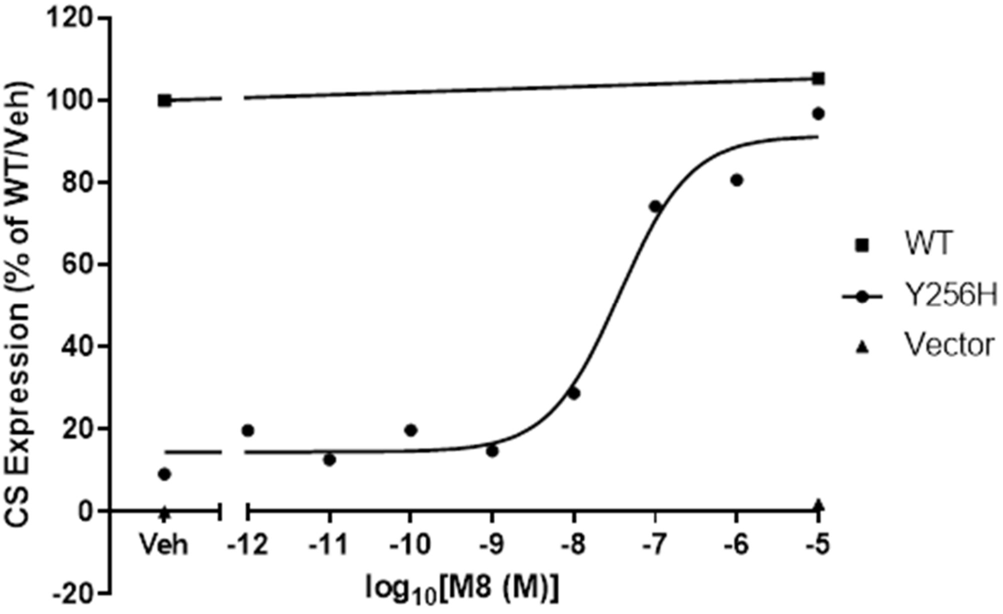

Figure S3: M8 rescue is dose-dependent. A receptor ELISA, with intact HEK 293-T cells transfected with empty vector, WT NK3R or Y256H NK3R following treatment without (Veh) or with a range of concentrations of M8, was used to measure receptor cell surface (CS) expression. Data are expressed as percentage of WT (treated with vehicle) following subtraction of background signal (empty vector transfected cells) and are presented as the means from one biological repeat, in which each data point was performed in triplicate.

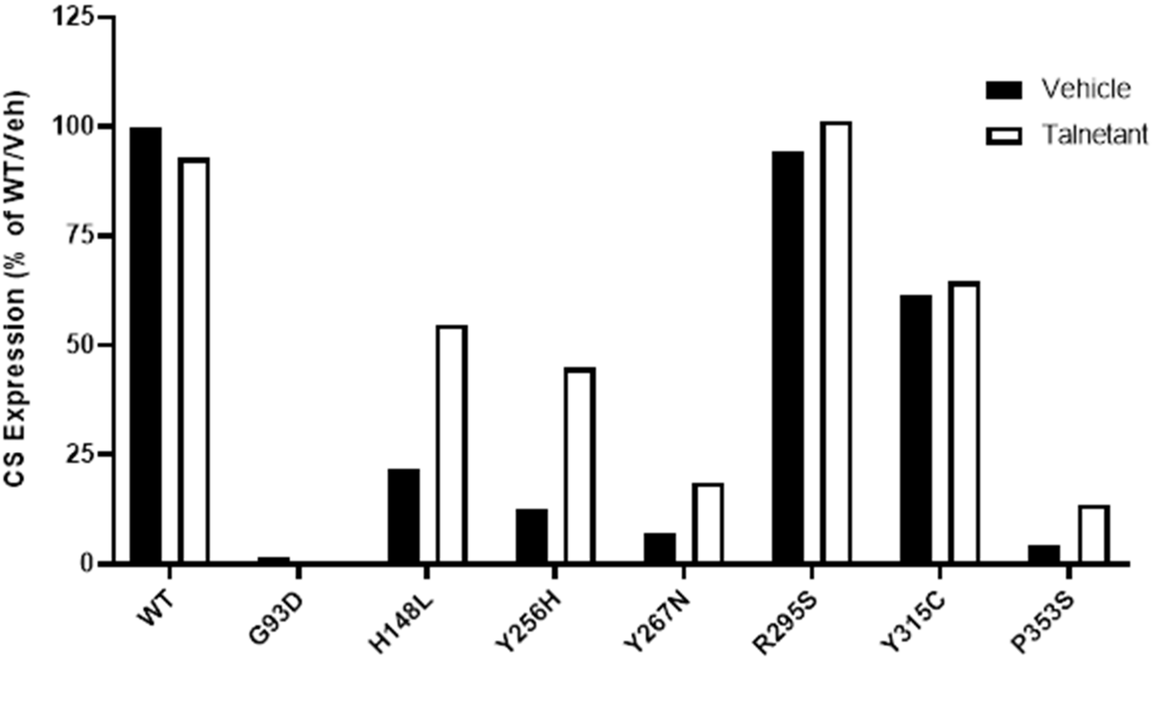

Figure S4: Talnetant is a PC for NK3R mutants. A receptor ELISA was used to measure receptor cell surface expression of wild-type (WT) or mutant NK3Rs following treatment with 1 μM talnetant or vehicle in HEK 293-T cells. Data are expressed as percentage of WT (treated with vehicle) cell surface expression following background subtraction (empty vector transfected cells) and are presented as the means from one biological repeat, in which each data point was performed in triplicate.

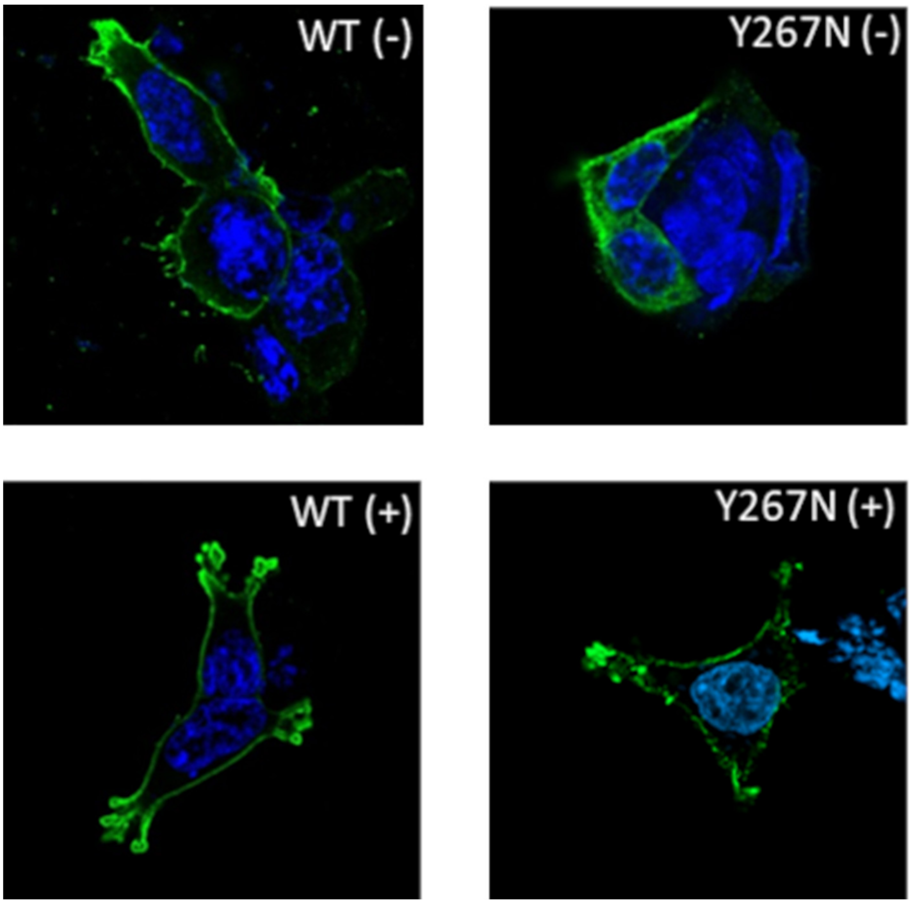

Figure S5: Intracellularly retained NK3R mutants can be rescued to the cell surface by M8. HEK 293-T cells expressing HA-tagged wild-type (WT) or mutant NK3Rs were treated with vehicle (-) (upper panels) or 1 μM M8 (+) (lower panels) for 16 h. HA epitopes were visualised with anti-HA primary and Alexa Fluor 488-conjugated secondary antibodies (green) and cell nuclei were stained with DAPI (blue). Images were taken with an oil immersed 63x objective on a Zeiss LSM800 confocal microscope.

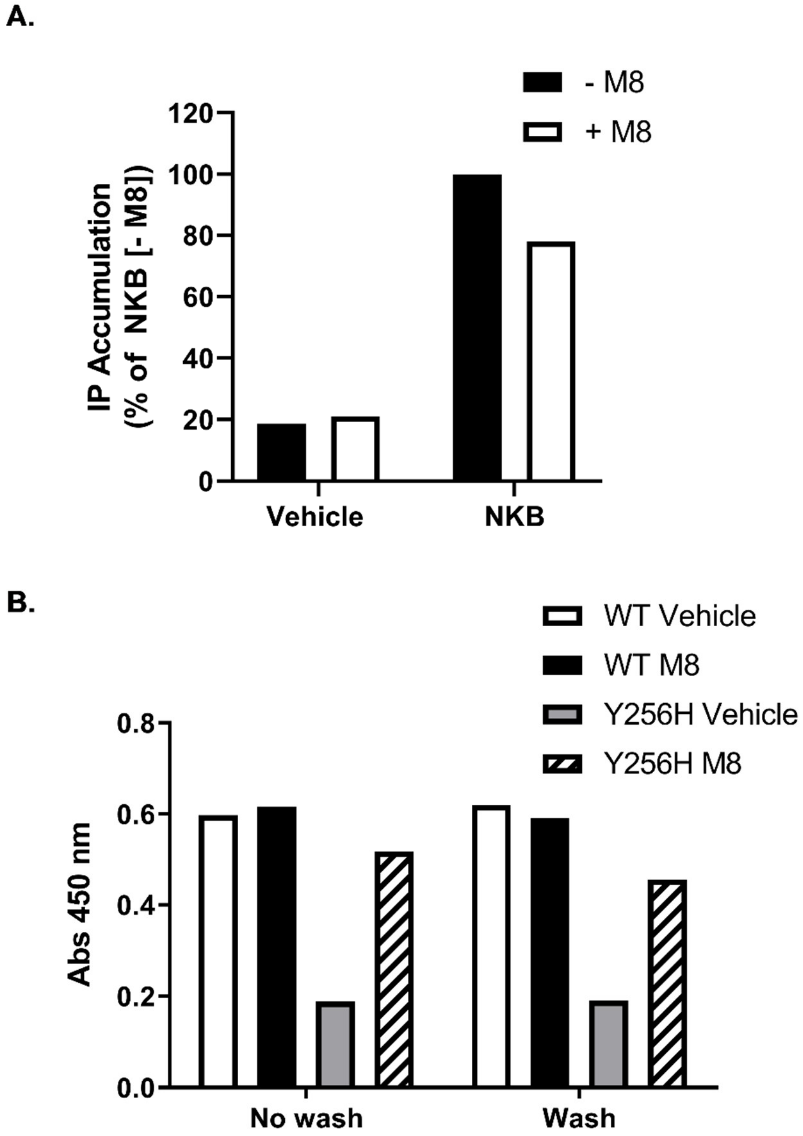

Figure S6: M8 wash-out protocol is effective and does not result in substantial loss of cell surface expression of Y256H NK3R mutant. (**A**) Receptor signalling was measured using an inositol phosphate accumulation assay. HEK 293-T cells expressing WT NK3R were pre-treated with 1 μM M8 (white bars) or vehicle (black bars) for 24 hours. Cells were then washed in buffer I (one on/off wash, incubation for three hours, and a final on/off wash), before treatment for one hour with vehicle (0.1% DMSO) or 100 nM NKB. Data are presented as the mean of three technical repeats expressed as a percentage of WT stimulated with NKB without prior treatment with M8 (n = 1). (**B**) HEK 293-T cells expressing wild-type (WT) or Y256H NK3R were treated with vehicle (0.1% DMSO) or 1 μM M8 for 24 hours. Cells were then washed in buffer I (one on/off wash, incubation for three hours, and a final on/off wash), prior to performing a receptor ELISA assay. Data are presented as the mean of three technical repeats, expressed as absorbance values following vector (baseline) subtraction (n = 1).
