# Supplementary material for: Functional Rescue of Inactivating Mutations of the Human Neurokinin 3 Receptor Using Pharmacological Chaperones
Source: Int J Mol Sci. 2022 Apr 21;23(9):4587. doi: 10.3390/ijms23094587 (PMC9100388; doi:10.3390/ijms23094587)
Supplement: Supplementary file 1 [file ijms-23-04587-s001.zip › Supplementary Methods.pdf]

Supplementary Methods

Radioligand Binding

COS-7 cells were seeded into 12-well plates and transfected with WT or mutant HA-NK3R using Lipofectamine2000 reagent (Invitrogen, Waltham, MA, USA). Cell culture media was changed after 6 h. Twenty-four hours after transfection, cells were treated with M8 (100 nM) or vehicle (0.01% DMSO) and further incubated for another 24 h. The M8 or vehicle treated cells were then washed twice with serum-free DMEM supplemented with 0.1 % BSA and incubated for 3 h. The cells were then washed with serum-free DMEM and incubated with 100,000 cpm of ^{125}I-NKB (Perkin-Elmer, Waltham, MA, USA). Cells were washed with ice-cold PBS three-times and then lysed with 0.2 M NaOH. Radioactivity was measured in a gamma counter.

To normalise for inter-assay variability, the sum of all data values for each biological repeat was calculated and each data point within that biological repeat was divided by this sum to calculate a ratio. Data were then expressed as a percentage of the values measured for WT HA-NK3R transfected cells (treated with vehicle) after subtraction of non-specific signal (measured in cells transfected with empty vector). Data were analysed using GraphPad Prism (Version 9) software (GraphPad Inc, San Diego, CA, USA) by one-way ANOVA followed by Dunnett's post-hoc test for comparison of mutant NK3Rs with WT with $p<0.05$ considered significant.

Biotinylation Assay

Plasma membrane proteins were isolated using a Pierce Cell Surface Protein Isolation Kit as described by the manufacturer (Thermo Fisher Scientific, Waltham, MA, USA). Briefly, HEK-293T cells were seeded into 6-well plates and transfected with WT or mutant HA-NK3R using Lipofectamine2000 reagent. Twenty-four hours after transfection, cells were treated with M8 (100 nM) or vehicle (0.01% DMSO) and further incubated for 24 h. The cells were washed with ice-cold PBS and then incubated with Sulfo-NHS-SS-biotin solution for 30 minutes at 4°C.

After quenching the biotinylation reaction, cells were harvested and washed with ice-cold PBS. Cells were then lysed using M-PER buffer (Thermo Scientific). After incubation for 30 minutes, cell debris was eliminated by centrifugation, and then the supernatant was incubated with NeutrAvidin Agarose bead solution for 1 hour. After three washes, biotinylated membrane proteins were denatured in 5X SDS-PAGE sample buffer for one hour at room temperature before immunoblotting. Na/K-ATPase antibody (Santa Cruz Biotech; sc-48345) was used for evaluation of membrane protein isolation.

Receptor ELISA with M8 Removal

HEK 293-T cells were plated at a density of 0.8×10^5 cells per well in Matrigel-coated 48-well plates. Twenty-four hours post-seeding, the cells were transfected with empty vector, WT HA-NK3R, or Y256H NK3R. Twenty-four hours post-transfection the cells were treated with 1 μM M8 or vehicle (0.1% DMSO), and incubated for a further 24 h. The cells were then washed in buffer I (DMEM supplemented with 20 mM HEPES and 0.1% BSA) to ensure removal of M8 (one wash, incubation for 3 h in buffer I and then a final wash). A receptor ELISA was performed on intact cells to quantify cell surface receptor expression with an antibody targeting the N-terminal HA-epitope tag, as previously described [14]. Data is presented as the mean of three technical repeats from one biological repeat.

Confocal Microscopy

HEK 293-T cells transfected with HA-tagged WT or Y267N mutant NK3R were grown on glass coverslips. Cells were fixed by incubation with 2% (w/v) paraformaldehyde in PBS for 15 min, followed by permeabilization with 0.1% (v/v) Triton X-100 in PBS. Coverslips were blocked with 2% (w/v) BSA and incubated with a mouse anti-HA antibody (HA-tag monoclonal antibody, #32-6700, Invitrogen, Waltham, MA, USA) followed by incubation with goat anti-mouse IgG (H+L) secondary antibody conjugated to Alexa Fluor 488 (#A-11001, Invitrogen, Waltham, MA, USA). Coverslips were mounted on microscope slides using mounting fluid with 4′,6-diamidino-2-phenylindole (DAPI) as an additive to visualise the nuclei (ProLong™ Diamond Antifade Mountant with DAPI, #P36962, Invitrogen, Waltham, MA, USA). Cells were imaged on a Zeiss LSM800 inverted confocal microscope using a 63x (NA 1.32) oil objective.
