# Supplementary figures and images for: Functional Rescue of Inactivating Mutations of the Human Neurokinin 3 Receptor Using Pharmacological Chaperones
Source: Int J Mol Sci. 2022 Apr 21;23(9):4587. doi: 10.3390/ijms23094587 (PMC9100388; doi:10.3390/ijms23094587)

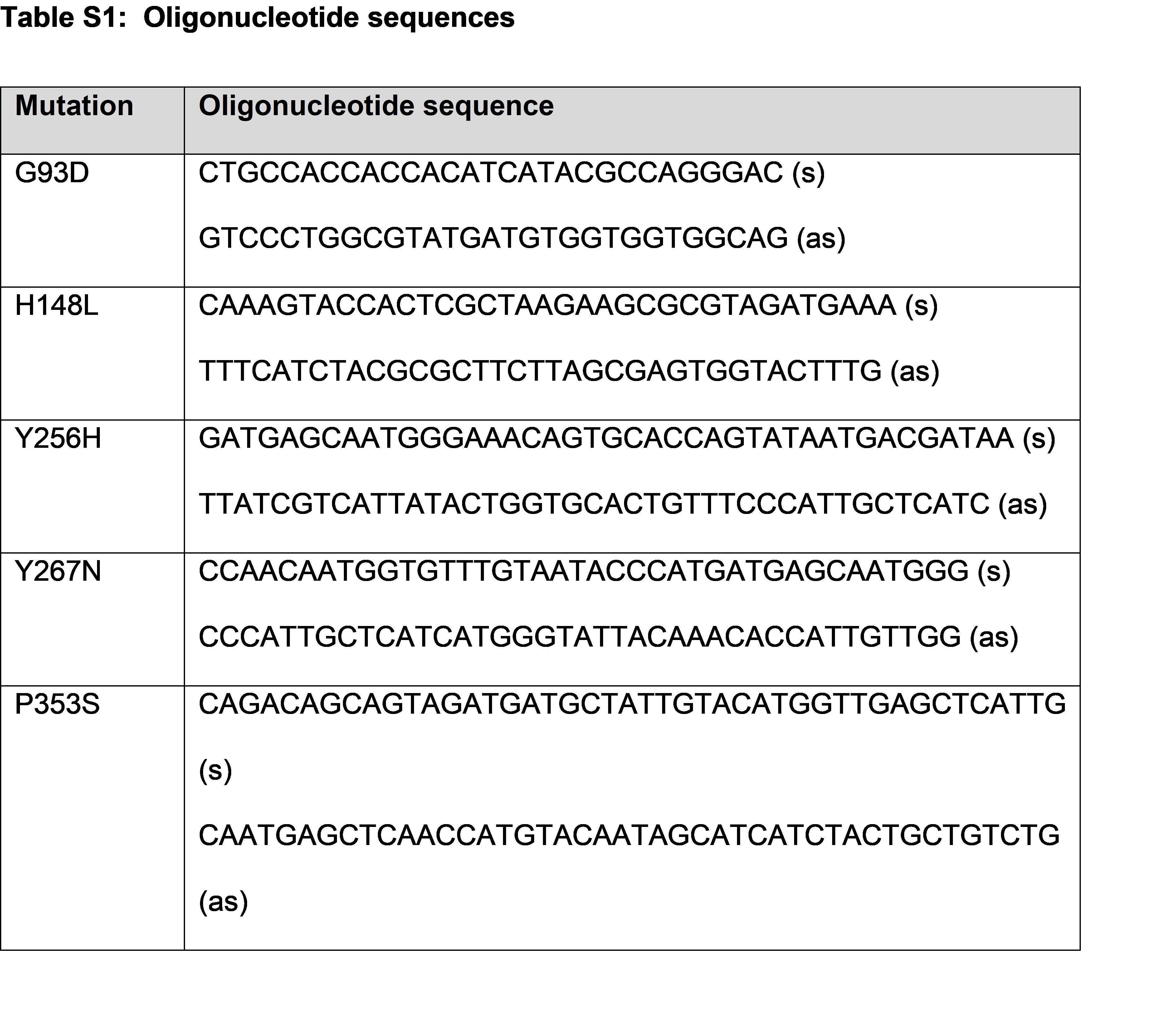

Supplement: Supplementary file 1 [file ijms-23-04587-s001.zip › Table S1.png]
